# A cobalamin-dependent pathway of choline demethylation from the human gut acetogen *Eubacterium limosum*

**DOI:** 10.1016/j.jbc.2025.108524

**Published:** 2025-04-23

**Authors:** Ruisheng Jiang, Duncan J. Kountz, Liwen Zhang, Joseph A. Krzycki

**Affiliations:** 1 Department of Microbiology, The Ohio State University, Columbus, Ohio, USA; 2 Campus Chemical Instrument Center Mass Spectrometry and Proteomics Facility, The Ohio State University, Columbus, Ohio, USA; 3 The Ohio State Biochemistry Program, The Ohio State University, Columbus, Ohio, USA

**Keywords:** bacterial metabolism, microbiology, energy metabolism, folate, microbiome, enzyme catalysis, cobalamin, one carbon metabolism, choline, acetogenesis

## Abstract

Elevated serum levels of trimethylamine N-oxide (TMAO) are reported to promote the development of atherosclerosis. TMAO is produced by hepatic oxidation of trimethylamine (TMA) produced by the gut microbiome from dietary quaternary amines such as choline. Net TMA production in the gut depends on microbial enzymes that either produce or consume TMA and its precursors. Here we report the elucidation of a novel microbial pathway consuming choline without TMA production. The human gut acetogen *Eubacterium limosum* grows by demethylating choline to N-N-dimethylaminoethanol. Quantitative mass spectral analysis of the proteome revealed a multi-protein choline to tetrahydrofolate (THF) methyltransferase system present only in choline-grown cells. The components are encoded in a gene cluster on the genome and include MthB, an MttB superfamily member; MthC, homologous to methylotrophic cobalamin-binding proteins; MthA, homologous to cobalamin:THF methyltransferases; and MthK, a protein related to serine kinases. Together, MthB, MthC, and MthA methylate THF with phosphocholine, but not choline or other quaternary amines. MthB specifically methylates Co(I)-MthC with phosphocholine. MthK acts as a bifunctional choline kinase which can utilize ATP or the MthB demethylation product, *N,N*-dimethylaminoethanol phosphate, to phosphorylate choline. Together, MthK, MthB, MthC, and MthA are proposed to carry out the methylation of THF with choline. These results outline a THF methylation pathway in which choline is first activated with ATP to phosphocholine prior to demethylation to form *N,N*-dimethylaminoethanol phosphate. The latter can be recycled by MthK to form more phosphocholine without expending additional ATP, thus minimizing energy utilization during choline-dependent acetogenesis.

The metabolic pathways employed by the gut microbiota can have dramatic impacts on human health. A notable example is the production of trimethylamine (TMA) from dietary quaternary amines (1, 2, 3). TMA produced by some members of the intestinal microbiota is absorbed into the bloodstream and subsequently oxidized to trimethylamine-N-oxide (TMAO) (Fig. S1) by a liver flavin-dependent monooxygenase (4). Impairment of the oxidation of TMA to TMAO can lead to the development of trimethylaminuria, a socially disabling disease, as TMAO is much less malodorous than TMA (5). However, serum levels of TMAO have been found to correlate with serious health issues. High TMAO levels correlate with an increase in plaque frequency and size in an atherosclerotic mouse model (1), as well as with increased risk of stroke and heart attack (6, 7, 8). High serum concentrations of TMAO further correlate with aggravation of graft *versus* host disease (9), increased risk of colorectal and liver cancers (10, 11, 12), as well as promotion of vascular inflammation (13). TMAO levels are predictive of increased risk of mortality for those suffering from kidney disease, coronary artery disease, or heart attack (8, 14, 15, 16).

Common quaternary amines found in the diet include L-carnitine (17, 18), γ-butyrobetaine (19), glycine betaine (20), and choline (21) (Fig. S1). TMA production from quaternary amines was first known to occur *via* conversion of quaternary amines to glycine betaine (22, 23, 24), which was then converted to acetylphosphate and TMA by GrdHI, glycine betaine reductase (25) (Fig. S1). More recently, oxygenase/reductases such as CntAB (26, 27), or YeaWX (3) have been described, which, respectively, produce TMA from L-carnitine or γ-butyrobetaine. L-carnitine is used by some enterobacteria as an electron acceptor, producing γ-butyrobetaine (28, 29, 30). The flavoprotein BbuA cleaves the CoA thioester of γ-butyrobetaine to crotonyl-CoA and TMA (31). Choline metabolism is considered a major source of TMA production in the gut (1, 32, 33). Choline is converted to TMA and acetaldehyde under anoxic conditions by CutC and CutD (Fig. S1), respectively, a glycyl radical choline:TMA lyase and the cognate activating protein (34, 35). The *cutCD* genes are widely distributed among bacteria, including representatives in the intestine (36). Serum choline concentration has been found to correlate with the severity of stroke (37) and the occurrence of coronary artery disease in a healthy population (38). Further, inhibitors of CutCD given to mouse models decreased net TMAO concentration as well as decreased net thrombosis potential (39), salt-induced hypertension (40), the circadian rhythm of energy cycling affecting obesity (41), and hepatic alcohol damage (42). The nutritional supplement glycerylphosphocholine has been shown to induce *cutCD* among the gut microbiota as well as promote atherosclerosis (43).

In recent years, the MttB superfamily of proteins has held promise as a means by which net TMA production in the gut might be lowered. The first described member of the family was the TMA methyltransferase MttB (Fig. S1) from *Methanosarcina barkeri*, which initiates methanogenesis by using TMA to methylate a corrinoid protein, which is subsequently used to methylate CoM, forming the ultimate precursor to methane (44). Subsequent sequencing of the *mttB* gene revealed a novel gene containing an in-frame amber codon (45), which was later found to be translated as the 22nd amino acid pyrrolysine (46, 47, 48, 49). Recent structural evidence indicates a key role of pyrrolysine in the active site of MttB (50). Gut methanogens such as *Methanomassiliicoccus* spp. possess MttB and have been proposed as a means of controlling intestinal TMA production (51).

Most members of the MttB superfamily are encoded by genes lacking the pyrrolysine codon leaving the function of the non-pyrrolysine MttB family members an open question. However, the pyrrolysine residue in MttB has been hypothesized to form an adduct with trimethylamine, forming a quaternary amine (46, 49, 50, 52, 53). This idea led to the further hypothesis that members of the MttB superfamily lacking pyrrolysine might directly demethylate quaternary amines, an idea subsequently proven when the MttB homolog MtgB from *Desulfitobacterium hafniense* was found to be a glycine betaine methyltransferase (54).

The genome of the intestinal acetogen *Eubacterium limosum* ATCC 8486 encodes 42 MttB superfamily members lacking pyrrolysine. Acetogens such as *E. limosum* can catabolically synthesize acetate and butyrate (55, 56, 57) from methyl-THF and carbon dioxide *via* the Wood-Ljungdahl pathway (58). *E. limosum* thus can grow by demethylation of quaternary amines such as glycine betaine, or choline (59), proline betaine, L*-*carnitine, and γ-butyrobetaine (56, 57, 60). MttB superfamily members MtpB, MtcB, and MtyB were, respectively, most abundant when grown on proline betaine, L-carnitine, or γ-butyrobetaine. Each protein was subsequently shown to methylate the Co(I)=corrinoid protein MtqC with the respective growth substrate. MtqC was demethylated by MtqA for the methylation of THF (56, 57, 60, 61). (Fig. S1).

Recently, Borton *et al.* (62) enumerated genes in the human microbiome encoding enzymes producing TMA from quaternary amines (such as *cutC, cntA, and yeaW*) as well as enzymes that do not produce TMA while metabolizing quaternary amines or TMA itself (*i.e.* genes encoding MttB superfamily members with or without pyrrolysine). Using a publicly available database assembled by Jie *et al.* (63) it was shown that diversity and abundance of these different genes could predict whether a particular microbiome was from a sufferer of atherosclerotic cardiovascular disease or a healthy control. The accuracy was not significantly different from employing the traditional lipid biomarkers commonly used to detect atherosclerosis.

These data demonstrate the necessity of further exploration of the metabolic functions of MttB superfamily members. Previously, no enzyme has been described that can directly demethylate choline (33). Over 40 years ago, the Gottschalk laboratory reported that several strains of *E. limosum* could grow by demethylating choline to N,N-dimethylaminoethanol (DMAE) with the production of acetate and butyrate and without the production of TMA (59). Here, we elucidate a pathway by which *E. limosum* demethylates choline and produces methyl-THF in this gut acetogen. In contrast to our previous findings with other quaternary amines, modification of choline was required to convert choline into a substrate of the MttB superfamily member MthB (Fig. 1) before demethylation.

**Figure 1:**
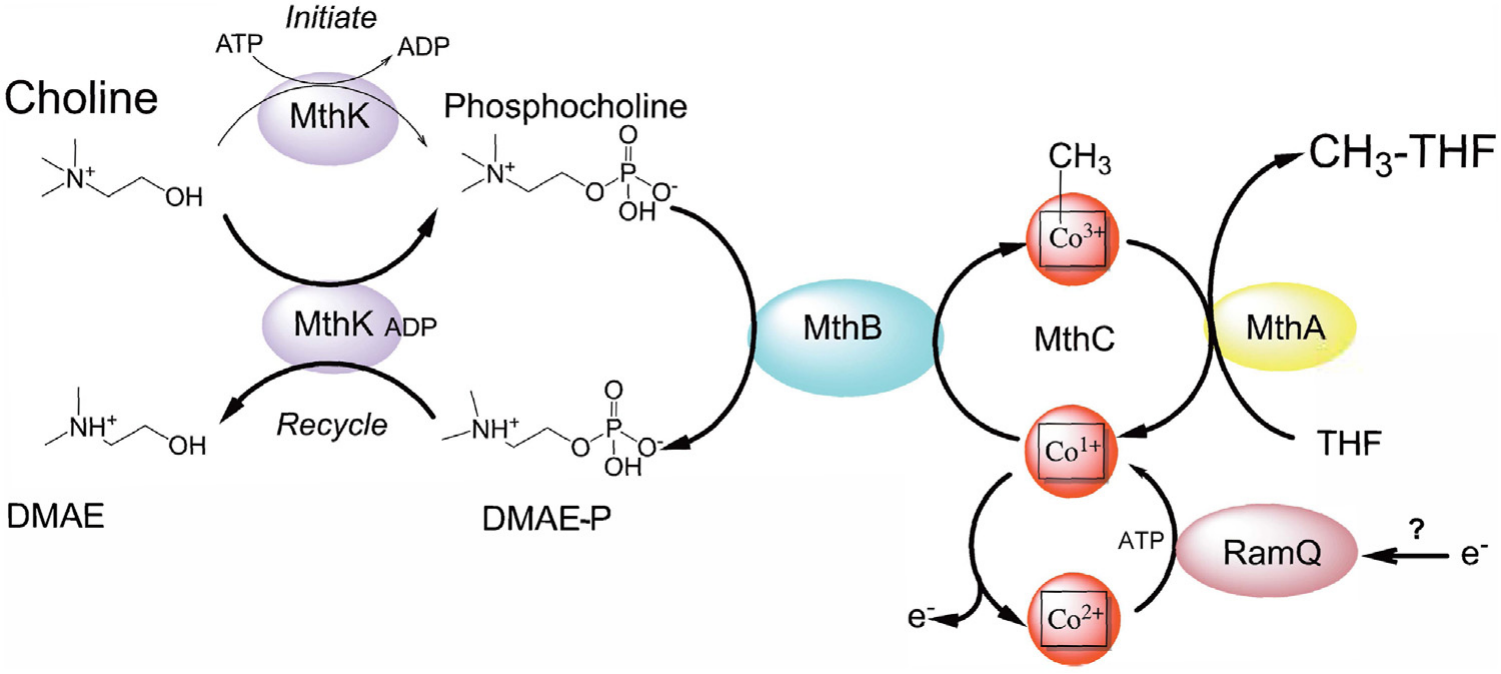
**Proposed pathway for the methylation of THF with choline by *E. limosum*.** To act as a substrate for MthB, choline must first be phosphorylated. To initiate choline demethylation, MthK utilizes ATP to provide phosphocholine, which would be demethylated by MthB for the methylation of Co(I)-MthC. As the reaction continues, dimethylaminoethanol phosphate (DMAE-P) can be recycled to produce phosphocholine for further cycles of demethylation. Methyl-Co(III)-MthC can be used by MthA to methylate tetrahydrofolate (THF) to regenerate Co(I)-MthC which can be remethylated by MthB. Following adventitious oxidation of Co(I)-MthC to Co(II)-MthC, RamQ with ATP and an unknown physiological electron source can bring Co(II)-MthC back into the catalytic cycle. In our *in vitro* assays RamQ mediates reduction of recombinant Co(II)-MthC to the active redox state with ATP and Ti(III)citrate.

## Results

### Growth of *E. limosum* on minimal media supplemented with choline

**Figure 2:**
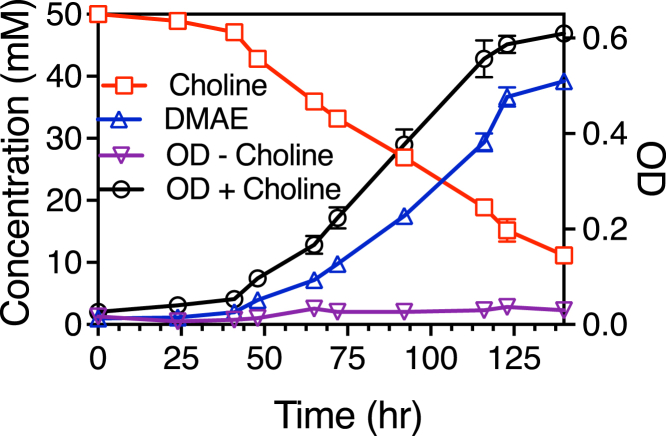
**Growth of *Eubacterium limosum* with concomitant demethylation of choline to DMAE (dimethylaminoethanol).** Growth was monitored by optical density (OD) at 600 nm of cultures in the absence or presence of 50 mM choline. Sample supernatants were obtained from the choline cultures at the indicated timepoints and both choline and DMEA were quantified by ^1^H-NMR. Each point on the graph represents the mean value from three independent cultures. Error bars represent standard deviation.

Since the first report of choline demethylation by *E. limosum* in 1981 (59), there has been no further description of the activity in this organism. Further, that seminal work focused on growth with glycine betaine, and the demethylation of choline was mentioned only in text without further documentation. Therefore, we set out to document the correlation of growth of *E. limosum* ATCC 8486 with choline demethylation. Initially, cultures supplemented with choline grew relatively slowly and after a long lag time, but after several transfers cultures achieved a growth rate that was maintained in subsequent culture. Growth was then measured in triplicate cultures (three biological replicates) in defined medium supplemented with 50 mM choline and aliquots removed for analysis by ^1^H-NMR. Controls lacking choline did not show measurable growth, while those supplemented with choline grew with a consistent doubling time (22 ± 4 h, n = 3). Choline concentrations were observed to decrease during growth, as measured by integration of the singlet peak at 3.19 ppm which corresponds to the ((CH_3_)_3_-N^+^-) group of choline. In contrast, the singlet peak at 2.90 ppm corresponding to the ((CH_3_)_2_-NH^+^-) group of DMAE increased during growth (Figs. 2, S2). At the final timepoint, the amount of DMAE produced (39.2 ± 0.3 mM, n = 3) was not significantly different than the amount of choline consumed (38.9 ± 0.5 mM, n = 3), consistent with a single demethylation of choline during growth as observed for other quaternary amines utilized by this organism (56, 57, 60). We further determined from triplicate biological replicates that 8.1 ± 1.4 g cell dry weight were produced per mol choline consumed.

Supernatants from the cultures were also analyzed by LC-MS (Fig. S3) to confirm choline consumption and DMAE production. Prior to inoculation, analysis revealed a prominent ion corresponding to choline with *m/z* = 104.1073 (calculated *m/z* = 104.1070). Following growth, in addition to the choline peak a prominent peak with an *m/z* = 90.0918 was observed, essentially equivalent to that of the choline demethylation product, DMAE (calculated *m/z* = 90.0813).

### The proteome of choline-grown *E. limosum* reveals a possible choline:THF methyltransferase system

Anaerobic bacteria such as *E. limosum* gain relatively little energy from methylotrophic acetogenesis (64). To maximize growth rate, many catabolic enzymes are highly abundant in these microbes. This is especially true of the enzymes that utilize the growth substrate to form methyl-THF. We exploited this abundance previously to identify and vet enzymes mediating THF methylation with other quaternary amines such as L-carnitine, γ-butyrobetaine, and proline betaine (56, 57, 60). Therefore, we examined the proteome of *E. limosum* grown on choline to identify candidate proteins mediating the methylation of THF with choline (Tables S1, S2).

Four biological replicate cultures were grown on choline, and each was harvested at mid-log phase. The individual cell pellets were subsequently lysed, and the proteins were subjected to trypsin digestion. The peptides were then analyzed by LC-MS/MS and mapped onto the completely sequenced genome of *E. limosum* ATCC 8486. The mol % abundance of each was estimated using the individual emPAI value compared to the summed emPAI values of all identified proteins (65). The mol % abundance of proteins from the four biological replicates of choline-grown cells was averaged and compared to the previously obtained proteome of *E. limosum* ATCC 8486 grown with DL-lactate (57) to determine those proteins preferentially abundant in cells grown on choline.

We obtained relative abundance for approximately 1600 proteins from choline-grown cells, approximately the same number as previously quantified from cells grown on DL-lactate. Within the top 26 most abundant proteins from choline grown cells we identified three candidates that might be involved in the corrinoid-dependent demethylation of choline and methylation of THF. These include an MttB superfamily member, WP_341455952.1, which we designated MthB; a corrinoid protein homolog, WP_038350932.1, which we designated MthC; and a methylcorrinoid:THF methyltransferase, WP_038350933.1, which we designated MthA (Tables 1, S2). Each of these proteins was much more abundant in cells grown on choline *versus* those grown on DL-lactate. Additionally, MthB, MthC, and MthA had not been previously identified in any of the proteomes from cells grown on proline betaine (57), L-carnitine (56), or γ-butyrobetaine (60). At a mol % abundance of 2.67% MthB was by far the most abundant MttB superfamily member found in the choline proteome. Five other MttB homologs were detected in the proteome, but each of these had a mol % abundance of ≤0.007%.

**Table 1:** Choline:THF methyltransferase system components found in *E. limosum* soluble proteome

Name	Accession number	Mol % of total soluble protein in choline grown cells	Mol % of total soluble protein in lactate grown cells	Fold change^a^	*p* value
MthB	WP_341455952.1^c^	2.7 ± 0.70	0.00042 ± 0.00058	6400	0.0003
MthC	WP_038350932.1	5.6 ± 1.2	Not detected	≥56,000^b^	<0.0001
MthA	WP_038350933.1	0.76 ± 0.29	0.00027 ± 0.00057	2800	0.0018
MthK	WP_052237066.1	0.22 ± 0.060	Not detected	≥2200^b^	0.0003
RamQ	WP_038351871.1	0.031 ± 0.0059	0.0094 ± 0.0034	3.3	0.0007

a Ratio of mol % protein in choline-grown *versus* DL*-*lactate-grown cells.

b A lower limit of detection of 0.0001% of total soluble protein was used to estimate this value.

c Fomerly designated WP_038350935.1 at the National Center for Biotechnology Information.

**Figure 3:**
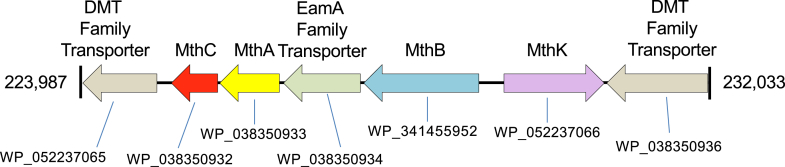
**Enzymes of the choline demethylation pathway are encoded in a gene cluster**. Colored arrows mark genes whose products are more highly abundant in cells grown on choline *versus* lactate. Genes in *grey* encode proteins that were not detected or at very low abundance in the soluble cell fraction subjected to proteomic analysis.

Examination of the *E. limosum* ATCC 8486 genome revealed that the genes encoding MthB, MthC, and MthA were located adjacently and on the same DNA strand (Fig. 3), suggestive of an operon structure and in keeping with their induction together during growth on choline. Between the *mthB* and the contiguous *mthA* and *mthC* genes is a gene encoding WP_038350934, a member of the EamA family of transporters. This protein, when searched against the Transporter Classification Database (66), revealed as top hits MttP from *M. barkeri*, a putative trimethylamine transporter (44), and LicB from *Haemophilus influenzae*, a high-affinity choline transporter (67). The proteomic methods employed here target primarily soluble proteins and discriminate against integral membrane proteins, but nonetheless a trace amount of WP_038350934 (Table S2) was detected in choline grown cells, but not in any of the previously obtained proteomes of *E. limosum* grown on DL-lactate, proline betaine, L-carnitine, or γ-butyrobetaine (56, 57, 60).

### MthB methylates Co(I)-MthC with phosphocholine

The above considerations strongly indicated that MthB, MthC, and MthA would play a key role in the methylation of THF with choline. Therefore, we recombinantly expressed *mthB*, *mthC,* and *mthA* separately in *Escherichia coli* and purified the proteins by Ni affinity chromatography prior to SDS PAGE analysis (Fig. S4). MthC was produced as an apoprotein, and therefore, the protein was reconstituted with hydroxycobalamin using the same procedure employed previously for reconstitution of the MtqC holoprotein (56, 57). The estimated incorporation of cobalamin into the MthC holoprotein preparations used in this study were above 0.9 mol cobalamin/mol protein.

We then tested the ability of MthB to methylate Co(I)-MthC with various substrates. The reconstituted MthC corrinoid protein was reduced to the Co(II)-MthC form using Ti(III)-citrate in a cuvette having a 2% H_2_ in N_2_ atmosphere. The active Co(I) state, indicated by the strong absorbance peak centered at 386 nm, was then obtained by the addition of recombinant *E. limosum* RamQ. RamQ is an ATP-dependent reductive corrinoid activase we employed previously (56, 57, 60) to reduce the corrinoid protein interacting with MttB superfamily members. The abundance of RamQ was increased by several-fold in choline-grown cells relative to those grown on DL-lactate (Tables 1, S2), as previously observed with cells grown on other quaternary amines (56, 57, 60).

Although MthB is abundant only in cells grown on choline, we unexpectantly found that MthB could not methylate Co(I)-MthC with choline. We tested other quaternary amines, including glycine betaine, L-carnitine, or proline betaine and found none of these were substrates for MthB methylation of Co(I)-MthC, even at concentrations up to 100 mM.

**Figure 4:**
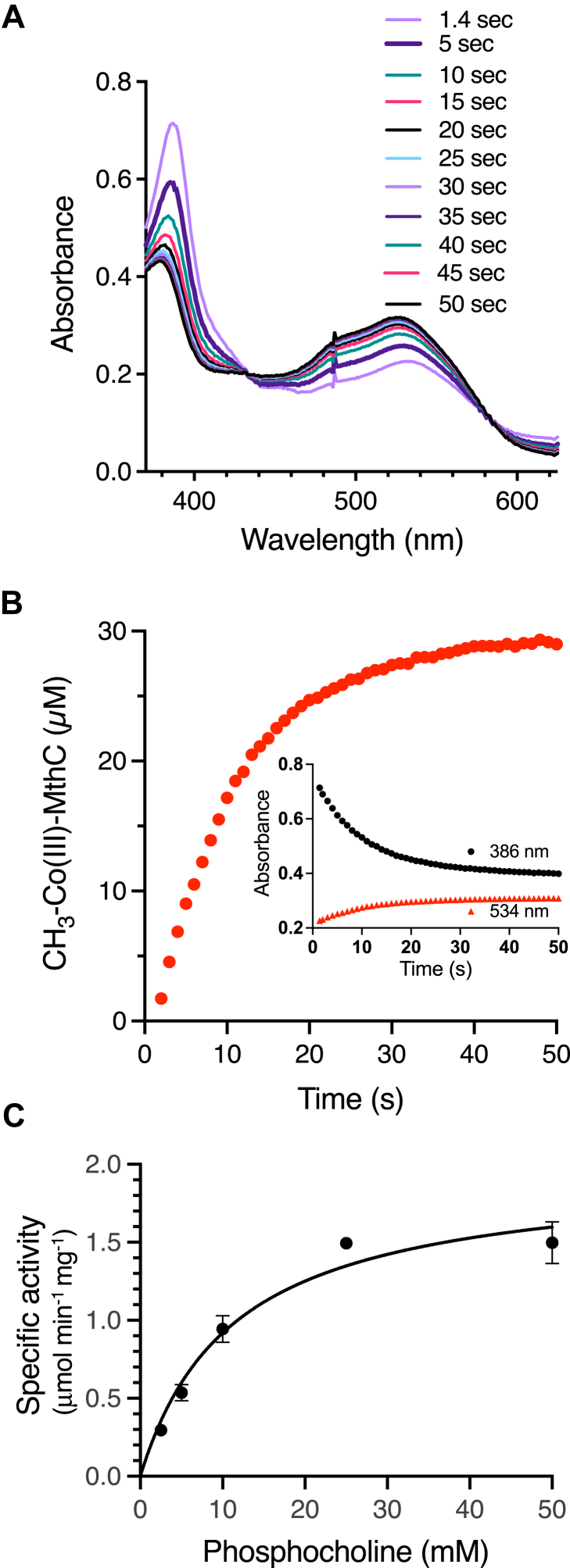
**MthB methylates Co(I)-MthC with phosphocholine**. *A*, spectra taken at the indicated times following initiation of the reaction by addition of 50 mM phosphocholine to a reaction mixture containing MthB and Co(I)-MthC generated by 2 mM RamQ with 2 mM Ti(III)citrate and 5 mM MgATP prior to initiation of the methylation reaction. The inset reveals the increase in absorbance at 534 nm used to monitor production of methyl-Co(III)-MthC and the decrease in absorbance at 386 nm corresponding to consumption of Co(I)-MthC. *B*, the production of methyl-Co(III)-MthC catalyzed by 1 μM MthB in the presence of 30 μM Co(I)-MthC in 50 mM phosphate buffer, pH 7.2. *C,* Kinetic analysis of the increase in MthB activity in the presence of increasing concentrations of phosphocholine. The data was fit to the Michaelis-Menten equation by non-linear regression using Prism 9.

We next examined MthB methyltransferase activity with choline derivatives that might be found in the gastrointestinal tract, and found that, while phosphotidylcholine, or glycerol phosphocholine were not substrates, MthB could methylate Co(I)-MthC with phosphocholine (Fig. 4*A*). Dependent on the presence of MthB and phosphocholine there was a sharp decrease in the absorbance peak at 386 nm concomitant with an increase in the absorbance at 534 nm indicating the conversion of Co(I)-MthC to methyl-Co(III)-MthC (Fig. 4*B*). The isosbestic points at 433 nm and 583 nm remained relatively stable, indicating the conversion of Co(I)-MthC to the methylated form occurred without the accumulation of significant amounts of a spectrally distinct form such as Co(II)-MthC (Fig. 4*A*). The rate of MthB methylation of 30 μM Co(I)-MthC was determined for three technical replicates with increasing amounts of phosphocholine and fit to the Michaelis-Menten equation (Fig. 4*C*). With phosphocholine and at 37 °C, MthB had an apparent *K*_M_ of 11.3 ± 1.9 mM and an apparent V_max_ of 2.0 ± 0.11 μmol min^-1^ mg^-1^ (*k*_cat_ of 106 ± 6 min^-1^). These values are comparable with those of MtpB, another MttB superfamily member, for methylation of its cognate corrinoid protein with proline betaine (*K*_M_ = 8 ± 2 mM, V_max_ = 3.1 ± 0.1 μmol min^-1^ mg^-1^ MtpB) (57).

### Methylation of THF with phosphocholine

**Figure 5:**
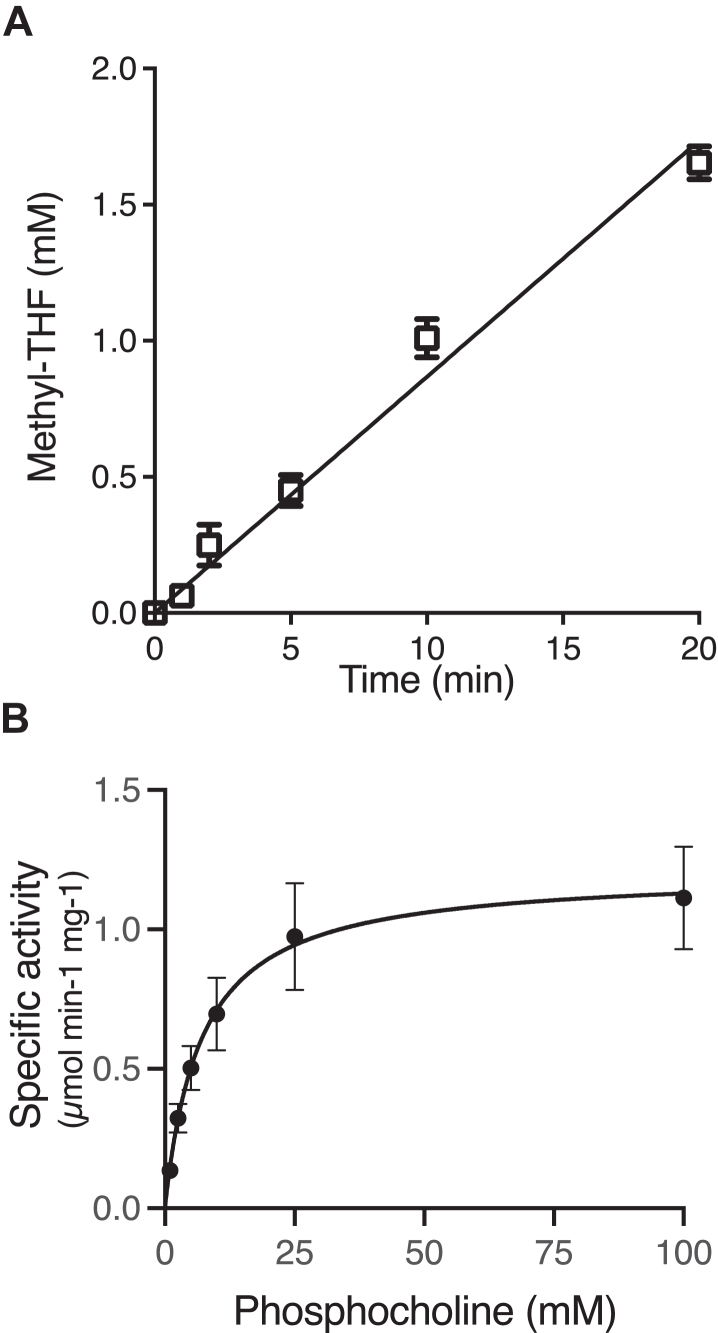
***In vitro* reconstitution of the phosphocholine:THF methyltransferase reaction.** MthB, MthC, and MthA and phosphocholine were preincubated under anaerobic conditions for 10 min with RamQ, ATP, and Ti(III)citrate to reduce MthC to the Co(I) state, and then the methylation reaction was initiated by addition of THF. *A*, methyl-THF formation in the presence of 50 mM phosphocholine. Each data point is the mean of methyl-THF formed in duplicate reactions with error bars representing range. No methyl-THF was detected in duplicate negative controls incubated for the same time but lacking either MthB, MthC, MthA, phosphocholine, or THF. *B*, kinetic analysis of the phosphocholine:THF methyltransferase reaction with rates determined at varying concentrations of phosphocholine. Points represent the mean initial rate of triplicate reactions with standard deviations shown by the error bars. Each line was fitted to the Michaelis-Menten equation using nonlinear regression.

Once a methyl group is removed from phosphocholine by MthB and MthC, it must be used to form methyl-THF for acetyl-CoA synthesis. Therefore, we next tested if the addition of MthA to MthB and Co(I)-MthC would allow methylation of the pterin cofactor. However, His-tagged recombinant MthA purified from *E. coli* was not stable at the 37 °C temperature employed for MthB activity assays and precipitated. Fortunately, we found the protein was stable at room temperature and therefore assays for THF methylation were carried out in a Coy Chamber maintained at 24 °C. In the presence of MthB and Co(I)-MthC, the further addition of MthA allowed the methylation of THF with phosphocholine as assayed by HPLC (Fig. 5*A*). The dependence of methyl-THF formation on increasing concentrations of phosphocholine was assessed with three technical replicates and fitted to the Michaelis-Menten equation by non-linear regression (Fig. 5*B*). The apparent *K*_M_ was 7.0 ± 1.3 mM while the apparent Vmax (expressed relative to MthA) was 1.2 ± 0.1 μmol min^-1^ mg^-1^ (*k*_cat_ of 37 ± 2 min^-1^).

### A choline kinase is present in the choline proteome

Given that phosphocholine was the only substrate utilized by MthB for methylation of Co(I)-MthB we tested the ability of choline-grown *E. limosum* to grow with phosphocholine, but this compound was not utilized as a growth substrate. Therefore, we examined the proteome of choline-grown *E. limosum* to determine how the growth substrate choline might be phosphorylated as a pre-requisite to demethylation. We identified WP_052237066.1, annotated in the genome as a phosphotransferase, as possible candidate for a choline kinase. This protein is present at 0.2 mol % in choline grown cells (Tables 1, S2), but was not detectable in the proteome from lactate-grown cells nor the proteomes of cells grown proline betaine (57), L-carnitine (56), or γ-butyrobetaine (60). Further, we found the gene encoding WP_052237066 is adjacent and divergently transcribed from *mthB*, *mthC*, and *mthA* in the *E. limosum* ATCC 8486 genome (Fig. 3). The Genbank record for WP_052237066.1 cites portions of the protein as having similarity to the catalytic site of serine/threonine protein kinases as well as an ATP binding site. A Phyre2 (68) alignment of LicA, a verified choline kinase from *Streptococcus pneumoniae* (69), with WP_052237066.1 revealed overall 32% similarity (19% identity). Residues of Brenner phosphotransferase motif and choline kinase motif of LicA were conserved in WP_052237066.1 (Fig. S5). Therefore, we recombinantly expressed this protein in *E. coli* (Fig. S4) and tested its ability to carry out phosphorylation of choline with ATP.

**Figure 6:**
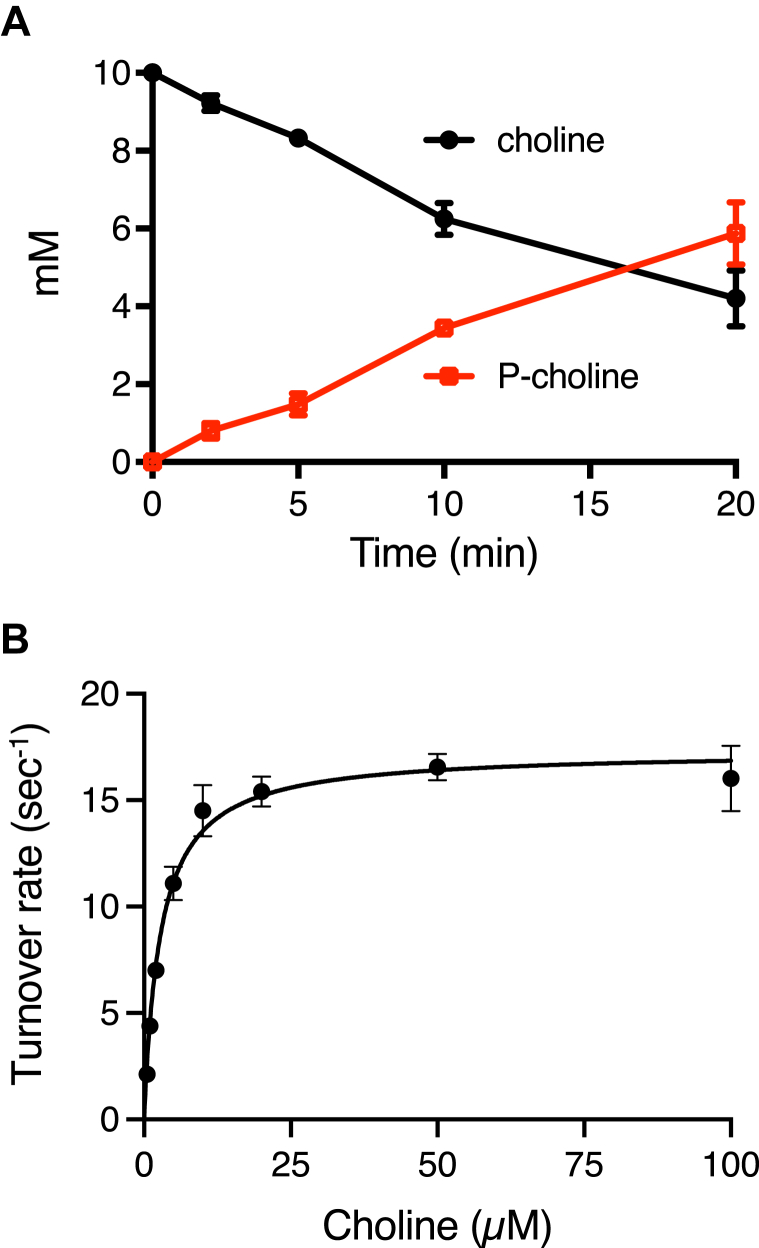
**MthK is a choline kinase**. *A*, MthK was incubated with MgATP and choline. At indicated timepoints triplicate reactions were quenched and choline and phosphocholine were determined by quantitative ^1^H-NMR. Error bars represent standard deviation. *B*, kinetic characterization of MthK using a spectral coupling assay employing pyruvate kinase and lactate dehydrogenase. Rates of choline phosphorylation were determined at increasing concentrations of choline at a constant ATP concentration. Points represent the mean initial rate of three reactions at each concentration with standard deviation shown by the error bars. Each line was plotted to the Michaelis–Menten equation using nonlinear regression.

We first confirmed the formation of phosphocholine *via* quantitative ^1^H-NMR analysis as described in Experimental Procedures. Timepoints were removed from three technical replicate reactions and concentrations measured by integration of the distinctive peaks corresponding to nine equivalent protons of the (CH_3_)_3_- groups of choline and phosphocholine. Under the conditions of this assay, choline was observed to decrease at a rate of 12 ± 0.6 μmol choline consumed min^-1^ mg protein^-1^ while phosphocholine increased simultaneously at a rate that was not significantly different, in keeping with the expected 1:1 choline:phosphocholine ratio (Fig. 6*A*). As WP_052237066 is part of the choline proteome, has choline kinase activity and is encoded divergently but immediately adjacent to the *mth* gene cluster (Fig. 3), we designated this protein as MthK.

We obtained the kinetic parameters of the MthK choline kinase using a coupled enzyme assay in which choline-dependent ADP production could be monitored by NADH oxidation in presence of pyruvate kinase and lactate dehydrogenase (Fig. 6*B*). Using this assay, MthK was found to have choline kinase activity. We measured initial enzyme activity of three technical replicates in the presence of different amounts of choline with the ATP concentration kept constant at 5 mM. When the data was fitted to the Michaelis-Menten equation by non-linear regression (Fig. 6*B*), the enzyme was found to have an apparent *K*_M_ of 2.8 ± 0.2 μM choline and apparent V_max_ of 17.3 ± 0.4 μmol min^-1^ mg protein^-1^ (*k*_cat_ = 13.8 ± 0.28 s^-1^) resulting in a *k*_cat_*/K*_M_ of 4900 s^-1^ mM^-1^. These parameters are comparable to documented choline kinases (Table S3).

### MthK acts as a DMAE phosphate:choline phosphotransferase

**Figure 7:**
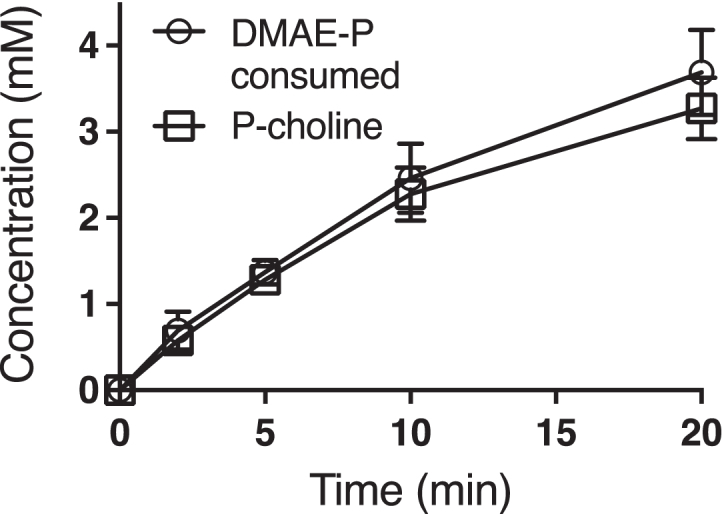
**MthK acts as a DMAE-P:choline phosphotransferase for the ATP-independent phosphorylation of choline**. MthK was incubated with 10 mM DMAE-P in the presence of 10 mM choline, 10 mM MgCl_2_, and 2 mM ADP. Samples were removed at indicated timepoints from triplicate reaction vials and the concentrations of DMAE-P and phosphocholine determined by ^1^H-NMR. No reaction occurred in the absence of ADP or choline.

It is thought that acetogens such as *E. limosum* gain approximately 0.6 mol ATP per mol of a methylotrophic substrate such as methanol (64). This called into question the ability of MthK to function in a catabolic pathway for demethylation of choline in *E. limosum* if ATP is invested each time phosphocholine is produced prior to demethylation by MthB. We hypothesized that this problem might be resolved if MthK could catalyze a phosphotransferase reaction between the demethylation product, DMAE phosphate (DMAE-P), and choline to produce phosphocholine in an ATP-independent manner. Employing quantitative ^1^H-NMR, we found that MthK could indeed act as a DMAE-P:choline phosphotransferase in the absence of ATP. (Fig. 7). The reaction required the presence of ADP as well as DMAE-P. The amount of phosphocholine made in reactions exceeded the amount of ADP added, confirming that the ADP acted catalytically in the phosphotransferase reaction. In the presence of 10 mM DMAE-P and 2 mM ADP, choline was phosphorylated at a rate of 4.4 ± 0.4 μmol min^-1^ mg MthK^-1^ or a turnover of approximately 210 min^-1^ as measured for three technical replicates. These results suggest that the overall phosphocholine demethylation pathway could be initiated with ATP but continue under cellular conditions with phosphate transfer from the intracellular pool of the demethylation product DMAE-P (Fig. 1).

### Genes encoding MthK and MthB homologs co-localize in many bacterial genomes

Bacteria are well-known to co-localize genes for a common cellular function. In *E. limosum* choline demethylation appears to require the activity of both MthK and MthB, whose genes are divergently transcribed from each other. As this is the first example to our knowledge of an enzyme that modifies a substrate prior to demethylation by an MttB superfamily member, we sought to determine if other bacteria adjacently encoded homologs of MthK and MthB by BLAST searches with MthK against cultured bacteria (Table S4) or bacterial sequences obtained from environmental samples (Table S5). We then examined each *mthK* homolog to determine if an *mthB* homolog was encoded within an adjacent three genes. We found such co-localization occurred in Firmicutes, particularly those of classes Clostridia and Negativicutes, as well as Gram-negative delta-proteobacteria. Other than *E. limosum*, three of these were previously shown to grow on choline (70, 71, 72). Furthermore, many environments contain bacteria whose genomes bear co-localizing genes encoding homologs of MthB and MthK.

## Discussion

Choline is considered among the most important substrates for gut microbiome TMA production. Not only is choline provided in the diet, but it is also supplied in the bile acid produced by most vertebrates and supplied to the gastrointestinal tract (73). To our knowledge, the MthB pathway of choline demethylation we have demonstrated here is the first microbial metabolic pathway that can operate under the anaerobic conditions that prevail for most microbiota in the human gut as an alternative to choline-dependent TMA production. By this novel choline pathway, *E. limosum* brings about the demethylation of choline and the subsequent methylation of THF (Fig. 1), thus forming a key metabolite required for subsequent synthesis of the major catabolic products acetate and butyrate.

MthB is now the sixth member of the very large MttB superfamily of proteins to have a distinctly identified physiological substrate, phosphocholine. Other known substrates of the MttB superfamily identified to date have been quaternary amines bearing a carboxylic acid group (54, 56, 57, 60). It is likely that the carboxylic acid moiety provides a key binding determinant for these MttB superfamily members. This may provide insight into why MthB cannot utilize choline directly. We hypothesize that recruitment of a choline kinase to the pathway allowed conversion of the alcohol moiety of the choline substrate, likewise to an acidic quaternary amine. This may have allowed an MthB precursor fewer evolutionary steps toward binding of a phosphate moiety *versus* those required toward accommodating an alcohol group.

MthK is a robust choline kinase. The *K*_M_ for choline of is one of the lowest reported for bacterial or eucaryotic choline kinases (Table S3) (69, 74, 75, 76, 77), while the *k*_*cat*_ for choline phosphorylation is easily within the range to supply the relatively high interior concentrations of phosphocholine required for formation of methyl-THF. Furthermore, once the pathway is initiated, MthK provides an avenue by which choline can be phosphorylated without the expenditure of ATP (Fig. 1). The direct product of MthB demethylation of phosphocholine, DMAE-P, can be used by MthK to phosphorylate choline. The dependence of this reaction on ADP suggests this cofactor provides the enzyme with a site to transiently phosphorylate during the transfer of the phosphoryl group from DMAE-P to choline. ADP might also act as an allosteric regulator of the DMAE-P:choline phosphotransferase and/or choline kinase activities. Further kinetic studies may help resolve these possibilities. In either case, the documented energy charge of *E. limosum* growing on methanol (78) indicates that the cellular concentrations of ADP and ATP may favor the ADP dependent DMAE-P:choline phosphotransferase reaction. ATP varies from 2 to 1 mM, while ADP remains constant at 1 mM during early to later stages of growth, assuring ample ADP for the DMAE-P:choline phosphotransferase reaction. This mechanism further solves the issue of dephosphorylating DMAE-P prior to expulsion from the cell as a primary catabolic product. The kinetics of MthK while mediating these two different routes to phosphocholine will be a worthwhile avenue for future study.

The molar growth yield on choline that we determined for *E. limosum* is consistent with ATP being used to initiate the pathway of choline demethylation, with the majority of choline utilized phosphorylated using DMAE-P generated by the demethylation reaction (Fig. 1). Using a bacterial Y_ATP_ value of 10.5 g cell dry weight per mol of ATP (79) our estimate of 8.1 ± 1.4 g dry cells/mole of choline would indicate the organism gains approximately 0.8 ± 0.2 mol ATP per mol choline. This estimate is close to the estimate of the 0.6 mol ATP formed per mol methanol for *E. limosum* (64), further supporting the hypothesis that ATP usage is minimized by the ATP-independent path of phosphocholine formation by MthK.

MthB is the most abundant MttB superfamily member in the proteome of *E. limosum* grown in our defined choline medium, and only four other MttB homologs could be detected whose relative abundance was several orders of magnitude below that of MthB (Table S2). We found analogous results in our previous work with proline betaine, L*-*carnitine, or γ-butyrobetaine (56, 57, 60). In contrast, a recent study of the proteome of *Eubacterium maltosivorans* grown on glycine betaine or carnitine medium with yeast extract was shown to produce a greater number of MttB homologs, with the most abundant being only 30-fold less than the most abundant (80). This discrepancy may lie in the growth medium. In our studies, we have grown *E. limosum* in defined medium lacking yeast extract. In preliminary studies, we found that the addition of yeast extract lowered the relative abundance of MthB to other detectable MttB family members in *E. limosum*^1^. Yeast extract is known to possess different quaternary amines (81, 82, 83) and thus can provide alternative substrates for demethylation by *E. limosum*. Nonetheless, it is notable that the proteins found in highest abundance following *E. maltosivorans* growth on carnitine (80) is 99% identical to MtyB, the *E. limosum* enzyme predominant during growth on γ-butyrobetaine, which we have documented can demethylate both γ-butyrobetaine and L-carnitine (60). In contrast, *E. maltosivorans* did not utilize choline for acetogenesis, as incubations with this substrate led to no net production of acetate or butyrate (80). This is puzzling considering *E. maltosivorans* possesses a nearly identical homolog of *E. limosum* MthB. However, when exposed to choline *E. maltosivorans* expressed only MttB superfamily members that had at best 30% identity to MthB, which were apparently inactive with the choline substrate. We have observed that *E. limosum* can have a very long lag phase before beginning log growth on choline^1^, which may explain these results with *E. maltosivorans*.

The *mthB* and *mthK* genes are adjacent and divergently transcribed. Divergent transcription has been recently recognized as a common widespread feature among co-regulated genes in bacteria or archaea (84); in keeping with MthB and MthK abundance only in cells of *E. limosum* grown on choline. We found multiple bacterial isolates co-localize their genes encoding homologs of MthB and MthK (Table S4). Several of these organisms are known to utilize choline for methylotrophic growth including strains of *Desulfitibacter alkalitolerans* (71), *Sporomusa malonica* (72), and Candidatus *Formimonas warabiya* (70). Further, organisms that co-localized the *mthK* and *mthB* homologs on their genomes were found in a variety of different hosts (Table S5) including human, other mammals, bird, and insect guts or feces where choline is a potential bacterial substrate. Those microbes from mammalian hosts tended to have the highest identity to *E. limosum mthK* and *mthB*. Different environmental locales varying from anaerobic digestors to marine sediments also possessed microbes bearing co-localized *mthK* and *mthB* homologs on their genomes. These observations further support the functional link we described here between these two proteins and strongly indicate that the pathway of choline demethylation we have elucidated may have relatively wide distribution among physiologically and environmentally significant bacteria.

## Experimental procedures

### Cell cultivation and growth experiments

*E. limosum* ATCC 8486 was obtained from the American Type Culture Collection and cultured anaerobically in at 37 °C in the defined LS medium under a nitrogen atmosphere essentially as described previously (56). The medium was supplemented with choline chloride or sodium DL-lactate (Millipore/Sigma) at indicated concentrations. The organism was initially grown with sodium lactate as sole energy source prior to transfer to choline as the sole energy source. Cultures were passed at least six times with choline prior to growth and proteomic experiments. Growth was monitored by optical density at 600 nm. Aliquots were removed at timepoints and choline and DMAE concentrations were monitored by quantitative NMR as described below. The masses of choline and the product DMAE in medium and cultures were confirmed by LC-MS analysis of culture supernatant performed at Ohio State Campus Chemical Instrument Center (CCIC) Mass Spectroscopy facility employing a Thermo Quantiva Triple Quadrupole HPLC MS/MS System.

### Analysis of the proteome of choline-grown *E. limosum* ATCC 8486

A set of four replicate cultures (10 ml each) were grown on defined LS medium with 50 mM choline. Each culture was anaerobically harvested in mid-log phase. Proteins were extracted and precipitated from cell lysate with TCA and 6 μg subjected to trypsin digestion. Prior to MS/MS, samples were subjected to 2-D liquid chromatography (LC) separation. MS/MS data was acquired with an Orbitrap Fusion mass spectrometer. Full details of sample preparation, LC, and MS/MS and data processing have been described previously (56, 57, 60). Processed sequence information from the MS/MS data was searched using Mascot Daemon by Matrix Science version 2.5.1 (Boston, MA) and searched against databases of the proteins encoded in the genome of *E. limosum* ATCC 8486 (85) maintained at the National Center for Biotechnology Information as NZ_CP019962.1. Scaffold (Proteomic Software, Inc., Portland OR) was used for the assembly of search results and the calculation of emPAI values to estimate the mol % of each identified protein within the total set of identified proteins (65). Abundances of proteins in choline-grown cells were compared to the previously obtained proteome of *E. limosum* 8486 grown with lactate (56, 57, 60). Student's *t*test with Benjamini-Hochberg correction was performed using Scaffold to evaluate if the difference for certain proteins between growth conditions is significant (*p* < 0.05).

### Cloning, expression, and purification of recombinant proteins

The *ramQ* gene was expressed in *E. coli* SG13009 and RamQ purified as previously described (56). The *mthC* or *mthK* genes were amplified from *E. limosum* genomic DNA and inserted into pET26 plasmids between NdeI and XhoI sites using the polymerase incomplete primer extension (PIPE) method (86). For both *mthC* and *mthK* genes sequence encoding a C-terminal hexahistidine-tag was added using the existing sequence on pET26. Recombinant MthB and MthA were obtained using synthetic copies of the *E. limosum mthB* and *mthA* genes that were codon optimized for expression in *E. coli* (87). Sequence encoding a C-terminal hexahistidine-tag was also added to both genes during their synthesis by Invitrogen GeneArt Gene Synthesis Services (ThermoFisher Scientific). The final products were then inserted into the pET26 vector for expression in *E. coli*.

MthB, MthC, MthA, and MthK were produced aerobically in *E. coli* BL21(DE3) at room temperature with 100 μM IPTG induction for 4 h. Cell pellets were collected, chilled with liquid nitrogen and then stored at −80°C. Thawed cells were suspended in 50 mM MOPS buffer, pH 7.0 with 500 mM NaCl, 2 mM DTT and 10 mM imidazole, then lysed using a French pressure cell operated at 20,000 psi. The supernatant obtained by centrifugation at 27,000*g* was then purified by nickel affinity columns eluted with a 10 to 500 mM imidazole gradient. MthC and MthK were purified aerobically using HiTrap chelating HP column (GE Healthcare). MthC was produced as an apoprotein lacking the corrinoid cofactor, and was therefore reconstituted anaerobically with 1 mM cobalamin using the protocol previously described (56, 57). Unincorporated cobalamin was removed from reconstituted MthC holoprotein using Superose12 (GE Healthcare) in a Coy Anaerobe Chamber (Coy Laboratories, Inc. Grass Lake, MI). The MthC protein was analyzed using the dicyano assay (88, 89) which indicated greater than 90% cobalamin incorporation.

Cell pellets containing MthA or MthB were suspended in the same MOPS buffer but made anaerobic by evacuation/flushing with N_2_ then loaded into a French pressure cell in the Coy chamber for cell lysis. The supernatant obtained by anoxic centrifugation of the lysed cells was then loaded anaerobically onto HisTrap FF Crude columns (GE Healthcare) in the Coy chamber for recombinant protein purification. Purified MthK, MthC, MthB, MthA, and RamQ were analyzed by SDS PAGE (Fig. S4) and were stored anaerobically at −80 °C in 20% glycerol until used.

### Enzymatic activities of MthK

A coupling assay was used to establish the choline kinase activity of MthK (90). ADP produced by the ATP dependent phosphorylation of choline was detected *via* the production of pyruvate from phosphoenolpyruvate by pyruvate kinase. The rate of pyruvate production was monitored by the decrease in absorbance at 340 nm (ε_340_ = 6.22 mM^-1^⋅cm^-1^) due to the oxidation of NADH by lactate dehydrogenase. The 500 μl reaction was conducted in an aerobic masked quartz cuvette (Starna Cells, Inc.) initially containing 5 mM ATP, 10 mM MgCl_2_, 100 mM KCl, 5 mM phosphoenolpyruvate, 1 mM DTT, 0.2 mM NADH, 50 units pyruvate kinase, 50 units lactate dehydrogenase, 0.1 mg/ml BSA, 0.05 to 0.5 μM MthK and 1 to 1000 μM choline in 50 mM glycine-NaOH buffer, pH 9.0. The reaction was initiated with the addition of choline. The production of phosphocholine by MthK was confirmed by NMR. The 200 μl reaction contained 5 mM ATP, 10 mM MgCl_2_, 100 mM KCl, 1 mM DTT, 0.1 mg/ml BSA, 1.2 uM MthK and 10 mM choline in 50 mM Tris buffer, pH 8.0. Aliquots (25 μl) were removed at each timepoint and quenched by the addition of 6 μl of 100% trichloroacetic acid (TCA). After 10 min centrifugation at 16,100*g*, the supernatant was transferred to an NMR tube for analysis as outlined in the NMR analysis section below. A set of samples with known concentration of choline and phosphocholine were analyzed at the same time to confirm the accuracy of concentrations determined for choline and phosphorylcholine from the reaction timepoints.

The phosphorylation of choline with DMAE-P in the presence of catalytic ADP was assayed in vials containing 2 mM ADP (disodium salt, 98% pure), 10 mM MgCl_2_, 100 mM KCl, 1 mM DTT, 0.1 mg/ml bovine serum albumin,1.2 μM MthK, 10 mM DMAE-P (Toronto Research Chemicals**)**, 10 mM choline, and 50 mM Tris buffer, pH 8.0 in a total volume of 0.15 ml. At each time point, a 25 μl aliquot was removed and 6 μl of 100% TCA added to quench the reaction. After 10 min centrifugation at 16,100*g*, the supernatant was transferred to an NMR tube for analysis as described below. Standards with known concentration of choline, phosphocholine (TCI Chemicals), DMAE, or DMAE-P were analyzed simultaneously to quantify these metabolites.

### Methylation of Co(I)-MthC holoprotein by MthB

Rates of Co(I)-MthC methylation by MthB with phosphocholine were determined essentially as described for other MttB family methyltransferases and their cognate corrinoid proteins (56, 57, 60). Reactions were monitored at 37 °C in stoppered anoxic masked quartz sub-micro cuvettes of 1-cm path length (Starna Cells, Inc.). The complete 100 μl reaction mixture contained 2 mM Ti(III)-citrate, 2 mM ATP, 2 mM MgCl_2_, 2 to 5 μM RamQ, 30 μM MthC, 1 μM MthB and phosphocholine in 100 mM MOPS buffer, pH 7.2. Reaction mixtures were first assembled in the cuvettes with all components but the proteins in an anaerobic chamber (Coy Laboratories, Inc.) maintained with an atmosphere 2% H_2_ and 98% N_2_. The cuvettes were stoppered and removed from the chamber. Anoxic MthC was injected by Hamilton syringe where it was reduced by the Ti(III)citrate in the reaction mix to Co(II)-MthC. The mixture was then monitored by capture of the UV/Vis spectrum at 1 s intervals using an HP 8453 diode-array spectrophotometer.

Prior to methylation, Co(II)-MthC was reduced to the Co(I) state by injection of the ATP dependent corrinoid reductase RamQ. Complete reduction of MthC was confirmed by the decrease of 475 nm peak characteristic Co(II)-MthC and the increase in the peak centered at 386 nm due to the formation of Co(I)-MthC. Methylation of Co(I)-MthC with phosphocholine was initiated by injection of MthB and followed by the rise in the absorbance peak centered at 534 nm. The change in concentrations of methyl-Co(III)-MthC and Co(I)-MthC were determined using empirically determined extinction coefficients, respectively, Δε = 2830 M^-1^ cm^-1^ and Δε = −13,200 M^-1^ cm^-1^.

### Methylation of THF with phosphocholine by MthB, MthC, and MthA

Reactions were carried out in dim red light in an anaerobic chamber at 24°C with an atmosphere of 2% H_2_ in N_2_. Reaction mixtures containing 5 mM THF (Sigma-Aldrich), 5 μM MthB, 30 μM MthC, 2 μM MthA, 8 μM RamQ, 2 mM Ti(III)citrate, 2 mM ATP, and 2 mM MgCl_2_ in 100 mM MOPS buffer, pH 7.0. The reaction mixture was preincubated for 10 min to allow reduction of MthC to the Co(I) state. Reactions were initiated by the addition of substrate. At each time point, 25 μl aliquots were removed and each quenched with 6 μl of 100% TCA. After 10 min centrifugation at 16,100g for 10 min, the supernatants from the reactions were transferred to stoppered, anoxic vials and stored in darkness at −80 °C until analysis. THF and methyl-THF concentrations in samples were determined by HPLC as described previously (56, 57). Briefly, thawed aliquots were analyzed *via* reverse-phase chromatography using a 250 mm × 4.6 mm Varian Microsorb MV-100 C18 column (Agilent) on a Dionex UltiMate 3000 HPLC system (ThermoFisher Scientific). The column was equilibrated and eluted with 7% (v/v) acetonitrile in 30 mM potassium phosphate buffer, pH 3.0. Peak integration was performed using Dionex Chromeleon version 6.8 and quantitation based on standard curve prepared with CH_3_-THF (Sigma-Aldrich). Initial rates of THF methylation were determined with a range of substrate concentration (1–100 mM) and non-linear regression performed in Prism Graphpad 5.0 to fit to the Michaelis-Menten equation to determine kinetic parameters.

### Quantitative NMR analysis of DMAE, choline and phosphocholine

Prior to analysis, aliquots of culture supernatant or reaction mixtures were thawed and centrifuged again at 161,00*g* for 10 min at 4 °C to remove any remaining cells or precipitated proteins. Samples were then diluted either five-fold to the final volume of 525 μl (for analysis with 5 × 178 mm NMR tubes) or 10-fold to a final volume of 200 μl (for analysis with 3 × 178 mm NMR tubes) with a final concentration 10% D_2_O and 100 μM sodium trimethylsilylpropanesulfonate (DSS) (Sigma-Aldrich) in H_2_O.

1D ^1^H and 2D ^1^H-^13^C HSQC NMR spectra were conducted at 298 °K on a Bruker Avance III HD 800 MHz at the Ohio State Campus Chemical Instrument Center NMR facility. Proton NMR spectra were acquired using 1.28 s acquisition time, 2 s relaxation delay, and 64 total scans. The water suppression was achieved using excitation sculpting with gradients. 2D ^1^H-^13^C HSQC was acquired with a standard Bruker pulse sequence with phase-sensitive mode using echo/antiecho-TPPI gradient selection. The experiment parameters include ∼4 ms acquisition time in ^13^C dimension, ∼80 ms acquisition time in the ^1^H dimension, 1s relaxation delay, 16 scans, ^13^C GARP decoupling during acquisition, and data matrix of 2048 × 128. The experimental time was roughly 38 min for one data set. Standards with 1 to 50 mM of commercially available DMAE, DMAE-P, choline and phosphorylcholine (>98% purity) were analyzed under the same conditions. All NMR data were processed with Bruker Topspin 3.6.1 (Billerica, MA). The data were typically zero-filled one time in both ^1^H and ^13^C dimension prior the application of window functions, followed by Fourier transformation, phasing, and baseline correction. Chemical shifts were internally referenced to DSS at 0.00 ppm. All peak assignments were made based on standards employing commercially available compounds of >98% purity. Existing databases (https://academic.oup.com/nar/article/46/D1/D608/4616873) were also used to assist peak assignments and metabolite identification. The ^13^C and ^1^H NMR signal were obtained for each sample to confirm metabolite assignments. The concentration of a given chemical was estimated employing standards of known concentration and comparing the integral of characteristic methyl peaks of each compound in ^1^H NMR to that of DSS. The signals from the three methyl groups of choline were located at 3.19 ppm (^1^H) and 56.7 ppm (^13^C) while from phosphocholine these were 3.22 ppm (^1^H) and 62.3 ppm (^13^C). The signals from the two methyl groups of DEAE were located at 2.90 ppm (^1^H) and 45.6 ppm (^13^C) while for DEAE-P these were 2.94 (^1^H) and 45.6 ppm (^13^C).

### Statistical Analyses

A single technical analysis of each of the supernatant of four biological replicates (individual cultures) was performed for determination of OD, choline and DMAE during growth. For proteome analysis, a single technical analysis was separately performed on each of four biological replicates (individual cultures) on each growth substrate compared. Student's *t* test was employed with the Benjamini-Hochberg correction using Scaffold (Proteomics Software) to determine if protein levels differed between choline or lactate cultures with *p* < 0.023. considered significant. At least three technical replicates of all enzyme assays were performed with a single enzyme preparation. Km and Vmax values were calculated with at least three technical replicates of enzyme assays at a particular concentration of substrate using Prism (Graphpad Software Inc.). Throughout the manuscript means are presented ± the calculated standard deviation. All experiments were repeated at least twice.

## Data availability

The mass spectrometry proteomics data have been deposited to the ProteomeXchange Consortium *via* the PRIDE partner repository (91) with the project accession number PXD013806 and project DOI 10.6019/PXD013806 (for the previously obtained lactate dataset (57)) and with the project accession number PXD033467 and project DOI 10.6019/PXD033467 (for the choline dataset). All other discussed data can be found in the manuscript, supporting files, and cited references.

## Supporting information

This article contains supporting information (69).

## Conflict of interest

The authors declare that they have no conflicts of interest with the contents of this article.

## Footnotes

1 D. Kountz and J. Krzycki, unpublished observations.
