# Supplementary material for: A cobalamin-dependent pathway of choline demethylation from the human gut acetogen Eubacterium limosum[image]
Source: J Biol Chem. 2025 Apr 23;301(6):108524. doi: 10.1016/j.jbc.2025.108524 (PMC12155760; doi:10.1016/j.jbc.2025.108524)
Supplement: Supporting Information [file mmc1.pdf]

Supporting Information

A cobalamin-dependent pathway of choline demethylation from the human gut acetogen *Eubacterium limosum*

Ruisheng Jiang[1], Duncan J. Kountz[1#], Liwen Zhang[2], and Joseph A. Krzycki[1,3*]

From the: [1]Department of Microbiology, [2]Campus Chemical Instrument Center Mass Spectrometry and Proteomics Facility, [3]The Ohio State Biochemistry Program, The Ohio State University, Columbus, OH 43210

Running Title: *Methylation of tetrahydrofolate with choline*

In this supporting document:

Table S3.docx Comparison of MthK activity with previously characterized choline kinases.docx

Figs. S1 to S5

Separate supporting

Excel files for this manuscript include the following:

Table S1. Scaffold Protein Report for choline-grown cells.xlsx

Table S2. Averaged protein abundances from choline-grown versus lactate-grown cells.xlsx

Table S4. MthK/MthB homologs encoded by co-localized genes in cultured microbes.xlsx

Table S5. MthK/MthB homologs encoded by co-localized genes in environmental samples.xlsx

Organism	K_M (mM)	k_{cat} (sec^{-1})	References
*E. limosum**	0.0028	13.8	This work
Streptococcus pneumoniae+	0.15	5.1	(70)
Human (α2)	0.098	83.1	(75)
Saccharomyces cerevisiae	0.27	153	(76)
Caenorhabditis elegans (A-2)	1.6	74	(77)
Rat brain	0.014	29.5	(78)

*Apparent values with 5 mM ATP.

+Apparent values with 2 mM ATP.

Table S3. Comparison of MthK activity with previously characterized choline kinases. Reference numbers are those used in the main publication.

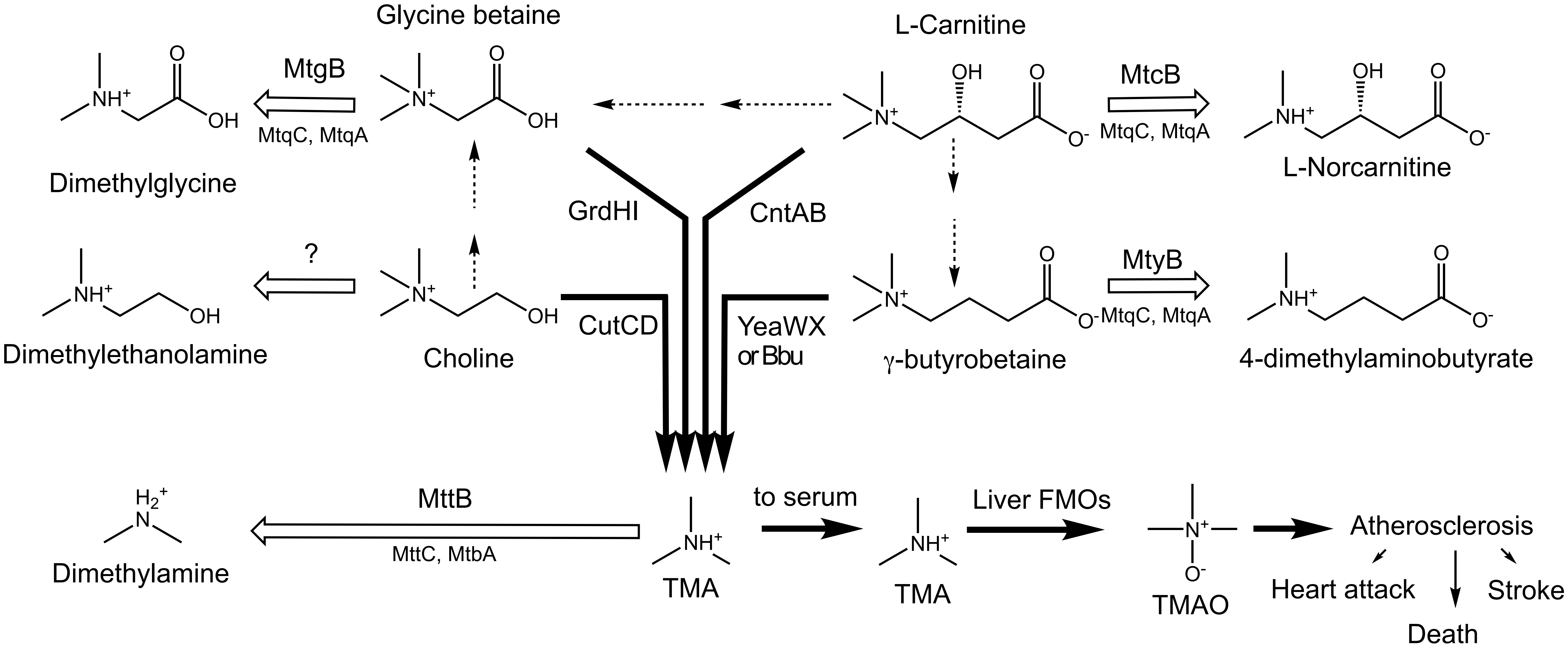

Fig. S1. Enzymes influencing net TMA production in the human gut. TMA may be produced via the oxygenases CntAB and YeaWX although oxygen is limiting in the intestine. Carnitine may also be converted to -butyrobetainyl-CoA and converted anaerobically by BbuA to TMA. Carnitine or choline can be converted to glycine betaine which can be used to generate TMA via glycine betaine reductase (GrdHI). Choline may be directly converted to TMA by CutCD. TMA can be converted to TMAO which correlates with severe health issues. Enzymes consuming TMA include the pyrrolysyl-protein MttB. *E. limosum* MttB superfamily proteins lacking pyrrolysine have been identified which can demethylate TMA precurosrs glycine betaine, L-carnitine, or γ-butyrobetaine without generation of TMA in order to provide methyl-THF for subsequent acetogenesis. Here we describe for the first time a pathway of choline demethylation which supplies methyl-THF for acetogenesis without the generation of TMA. For more details, see main text.

Fig. S1

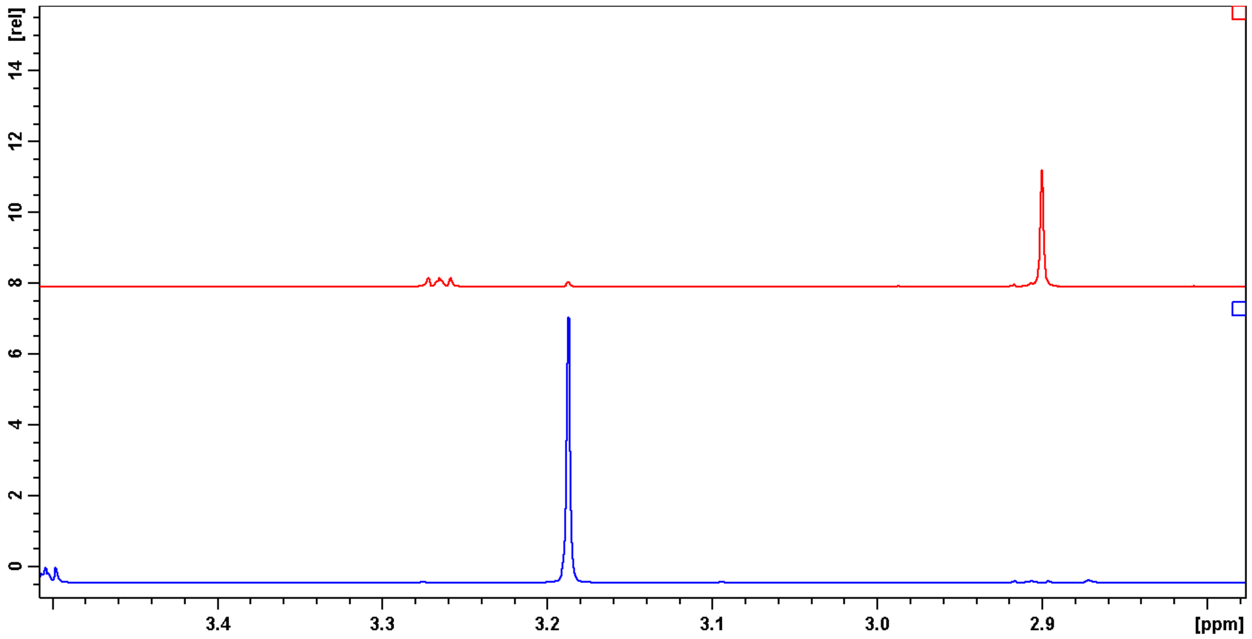

Fig. S2. ^{1}H-NMR spectra of the supernatants from an *E. limosum* culture prior to initiation of log phase growth (bottom spectrum) and following the end of growth (top spectrum). The predominant singlet peak at 3.19 ppm arising from the 9 protons of the 3 N-bound methyl groups in choline is observed prior to growth, whereas after growth and choline demethylation the singlet peak at 2.90 arising from 6 equivalent protons of the two remaining N-bound methyl groups of N-N-dimethylethanolamine (DEAE) is prominent.

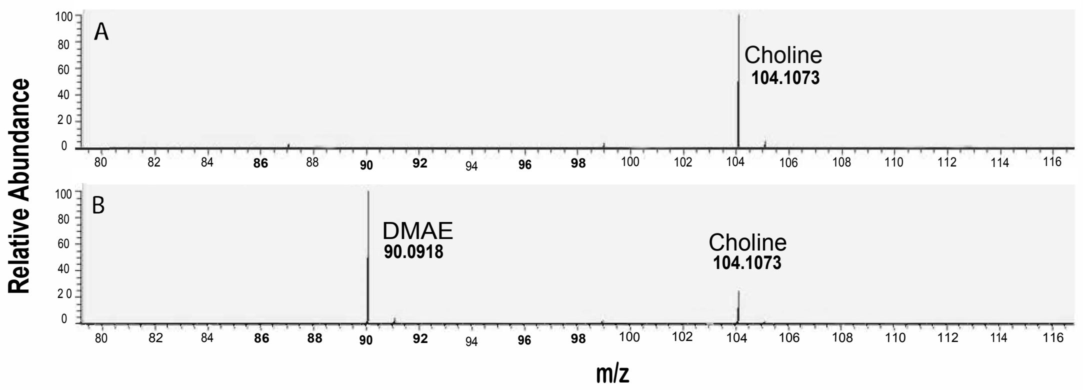

Fig. S3. Mass spectral analysis of choline culture supernatant removed (A) before the initiation of growth and (B) after the consumption of choline and production of DEAE.

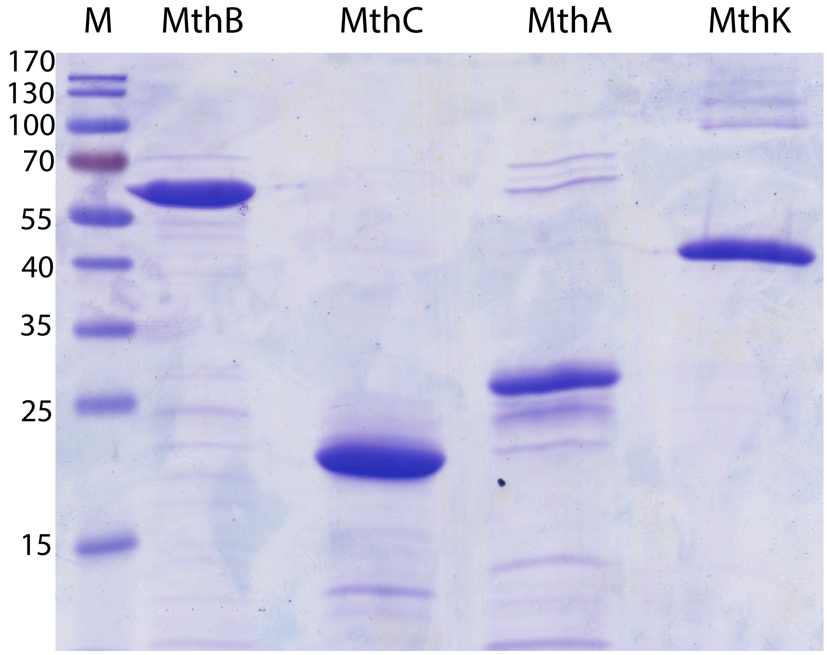

Fig. S4. SDS 12.5% PAGE gel of recombinant MthB, MthC, MthA and MthK preparations used in this study. The numbers to the left of the molecular markers lane (M) represent mass in kDa.

```
MthK_    1 MVDGKFIEMQTEIMKNYPPANAQALLNIFNAASGMQEYLDNVLAKTRESYLYTQLRDLVE
SpCK     1 ------------------------------------------------------MEKIIK


                           Ploop
MthK_   61 -NYYDIGTLLDVYQIFGGYINTSFGIYTEKNGKKQTWFVRKYKNGKE-LESLLFEHSMLK
SpCK     7 EKISSLLEVLSVEQL-GGMTNQNYLAKTTNK----QYIVKFFGKGTEKLINRQDEKYNLE
                              hc p              a


MthK_  119 YAKENGFSYGAVPIPAKDGKTYHEVTETTTEGETKSYFAVFNFVGGKAHYDWIPNWANAG
SpCK    66 LLKDLGLD-VKNYLFDIEAGI-----------------KVNEYIESAITLD-------ST
                                                    aaa    r

MthK_  179 VADLTVISAAKSLAEFHNSTRGFDPEGRHGDNIMDNEDITVNEIIRKFPKTLKEYRKSYA
SpCK   101 SIKTKFDKIAPILQTIHTSAKELR------------GEFAPFEEIKKYESLIEE------

                                                       Brenner
MthK_  239 EAGFENVYTEYYDANYDYFAKMCERSVIPDVDYDTMVSNVCHCDFHPGNFKYLDNGEVCG
SpCK   143 --QIPYANYESVRNAVFSLEKRLAD--------LGVDRKSCHIDLVPENFIESPQGR-LY
                                                      h   rp a

             CK motif
MthK_  299 SFDYDMAKIDSRLFELGLAIHYCFSSWLSDTNGIINLDRASLFVKTYDEELHKAGGIEPL
SpCK   192 LIDWEYSSMNDPMWDLAALFL----------ESEFTSQEEETFLSHYE---------SDQ
             p cq


MthK_  359 TDIEKKYLYEVTVQGALYDLGWCSSACVYDSTLDPYEYLFYTQHFVACLKWLEANEAAFR
SpCK   233 TPVSHEKIAIYKILQDTIWSLWTVYKEEQGEDFGDYGVNRYQRAVKGLASY---------
                             q  q             q


MthK_  419 KAFD
SpCK       ----
```

Fig. S5. Phyre2 generated structure based sequence alignment of MthK versus *Streptococcus pnuemoniae* choline kinase (SpCK) (1). Although the aligned portions of the two sequences have only 19% identity (black shading) and 13% homology (grey shading) both contain the Brenner motif characteristic of phosphotransferases and close variations of the choline kinase (CK) motif. Letters beneath SpCK residues indicate catalytic roles played by those residues in binding substrate (1); q, interacts with quaternary amine of choline; h, interacts with hydroxyl of choline; c, proposed to act in catalysis; a, interacts with adenine; r, interacts with ribose; and; p, interacts with alpha phosphate. Bolded italicized SpCK residues were shown important for catalysis by mutagenesis (1).

1. Wang, L., Jiang, Y. L., Zhang, J. R., Zhou, C. Z., and Chen, Y. (2015) Structural and enzymatic characterization of the choline kinase LicA from *Streptococcus pneumoniae* PloS one 10, e0120467 10.1371/journal.pone.0120467
